# Facilitators and inhibitors in hospital-to-home transitional care for elderly patients with chronic diseases: A meta-synthesis of qualitative studies

**DOI:** 10.3389/fpubh.2023.1047723

**Published:** 2023-02-13

**Authors:** Mengjie Sun, Lamei Liu, Jianan Wang, Mengyao Zhuansun, Tongyao Xu, Yumeng Qian, Ronnell Dela Rosa

**Affiliations:** ^1^School of Nursing and Health, Zhengzhou University, Zhengzhou, Henan, China; ^2^School of Nursing, Philippine Women's University, Manila, Philippines

**Keywords:** older patients, chronic diseases, transitional care, discharge, qualitative research

## Abstract

**Background:**

Chronic diseases are long-term, recurring and prolonged, requiring frequent travel to and from the hospital, community, and home settings to access different levels of care. Hospital-to-home transition is challenging travel for elderly patients with chronic diseases. Unhealthy care transition practices may be associated with an increased risk of adverse outcomes and readmission rates. The safety and quality of care transitions have gained global attention, and healthcare providers have a responsibility to help older adults make a smooth, safe, and healthy transition.

**Objective:**

This study aims to provide a more comprehensive understanding of what may shape health transitions in older adults from multiple perspectives, including older chronic patients, caregivers, and healthcare providers.

**Methods:**

Six databases were searched during January 2022, including Pubmed, web of science, Cochrane, Embase, CINAHL (EBSCO), and PsycINFO (Ovid). The qualitative meta-synthesis was performed following the Preferred Reporting Items for Systematic Reviews and Meta-Analyses (PRISMA) recommendations. The quality of included studies was appraised using the Critical Appraisal Skills Programme (CASP) qualitative research appraisal tool. A narrative synthesis was conducted informed by Meleis's Theory of Transition.

**Results:**

Seventeen studies identified individual and community-focused facilitators and inhibitors mapped to three themes, older adult resilience, relationships and connections, and uninterrupted care transfer supply chain.

**Conclusion:**

This study identified potential transition facilitators and inhibitors for incoming older adults transitioning from hospital to home, and these findings may inform the development of interventions to target resilience in adapting to a new home environment, and human relations and connections for building partnerships, as well as an uninterrupted supply chain of care transfer at hospital-home delivery.

**Systematic review registration:**

www.crd.york.ac.uk/prospero/, identifier: CRD42022350478.

## Introduction

With the development of an aging population, chronic disease health problems are becoming increasingly prevalent and the coexistence of multiple diseases is becoming more severe among older adults worldwide (1). Approximately 50% of older adults are reported to have two or more chronic diseases (2). Compared to a single chronic disease, older adults with multiple diseases are associated with poorer quality of life, higher risk of adverse drug events, repeat care, and death (3), increasing the social and medical burden. In this context, with national efforts to reduce the burden of health care, coupled with increased hospital turnaround efficiency and shorter hospital stays, older adults with complex health problems often have more complex needs, requiring multiple health care providers to provide a wide range of health and geriatric care equipment in multiple care settings, as well as frequent transitions between hospital and home (4, 5). Approximately 1 in 5 older patients experienced adverse events during hospital-to-home transitions, including unplanned readmissions within a month of discharge, medication errors, and even death (6–8). These adverse events were associated with uncoordinated, discontinuous transitions of care across healthcare settings (9–12). Improving the safety and quality of care transitions had become a global concern (13, 14).

Transition is defined as the process of changing from one state, or condition to another, which is a complex, multidimensional process that encompasses awareness, engagement, and period (15, 16). Assisting patients in managing life transitions is a key function of nursing (16). The Transition from hospital to home is often a ternary process involving patients, families, and health care providers, and the common goal for the stakeholders involved is that older adults are able to make healthy transitions after discharge from the hospital in accordance with established goals or are supported in problematic transitions of care. According to Meleis' transition theory, healthy transitions are characterized by response patterns that include process indicators (e.g., coping skills) and outcome indicators (e.g., wellbeing). And some transition conditions at the individual level (e.g., cultural beliefs, attitudes, readiness and knowledge related to transition), community level (community resources and support), etc., and social level (e.g., social norms) can facilitate or inhibit healthy transitions (15).

To promote healthy and safe patient transitions, transitional care models have been developed to bridge the gap between patients in different healthcare settings and different levels of care by providing continuous, coordinated care (5, 17). However, there is some controversy about the effectiveness of implementing transitional care interventions (18, 19). Although studies have shown that transitional care has the potential to improve health system efficiency and reduce adverse events and lower patient readmission rates (20, 21), difficulties remain in improving user experience aspects and satisfaction (22, 23). User experience can improve the process and quality of healthcare delivery (4, 24–26). Several studies have focused on user experience and satisfaction in care transitions, and have received some review attention (23, 27–29). The more recent are Hestevik et al. (23), Chen et al. (29), Høy et al. (28), and Joo et al. (27). Høy et al. (28), and Hestevik et al. (23) explored the perspective of older adults and Joo et al. (27) included family caregivers in addition to patients. Chen et al. (29) reviewed the barriers and facilitators of transition care for stroke patients and their caregivers. This was a review of specific diseases. In contrast, the prevalence of multimorbidity in the elderly population is 55–98% (30), requiring us to focus on a broader population of chronic disease, not just the transition of patients with specific diseases. Høy et al. (28) drafted a protocol that focused on exploring patient preferences, challenges, and levels of involvement in care transitions. However, the protocol was only planned to include literature published after 2010. The main results of the more important his review have not been reported. Hestevik et al. (23) examined the experiences of older adults adjusting to daily life at home after discharge from the hospital, synthesizing the results of 13 studies as (i) experiencing an insecure and unsafe transition, (ii) settling into a new situation at home, (iii) what would I do without my informal caregiver? and (iv) experience of a paternalistic medical model. Joo et al. (27) reviewed seven studies to understand the experiences and perceptions of patients with chronic illnesses and their caregivers as they transitioned from a medical setting to home. They reported that the transition from hospital to home was influenced by the following barriers and facilitators: communication with multiple healthcare providers, self-management, and psychological stress and family caregiver support and nurse-provided patient-centered care. However, the study population was patients ≥18 years of age and older with chronic conditions. None of these reviews included studies of health care workers' perceptions of transitions of care for older adults. Without a doubt, patient experience is important for the quality of care during the transition and is considered one of the three cores of healthcare quality (31). Yet the transition of care is a triadic process involving patients, families, and health care professionals. Healthcare professionals, especially nurses, as the primary implementers and providers of transitional care, need to interact with other health care professionals, family members, and others in the transition of care to assess the biological, psychological, social, and emotional needs of the patient, and their perceptions and experiences also influence the patient's health transition (24, 32, 33).

Researchers have not identified a published systematic review examining the transition conditions of older adults from hospital to home that has included older adults, caregivers, and health care providers. To achieve this goal, researchers need to understand what facilitates and inhibits the transition process from the perspective of older adults, their family caregivers, and health care providers. The study aims to address this question: a more comprehensive understanding of what facilitates or inhibits the transition of older adults transitioning from hospital to home from multiple perspectives, based on Meleis' transition theory, is important for an evidence-based approach to developing interventions for care transitions.

## Methods

### Design

This is a systematic evaluation and meta-synthesis of qualitative research. Qualitative synthesis follows the recommendations of the Preferred Reporting Items for Systematic Reviews and Meta-Analysis (PRISMA).

The study aimed to address the following question: What facilitates or hinders the transition for older adults from hospital to home? We used the SPICE framework (34) to formulate the review question. “Setting (where): healthcare facility, patient's home” “Perspective (for whom?): older adults transitioning from hospital to home, their caregivers, and health care providers” “Phenomena of interest: transition from hospital to home” “Comparison: not applicable” “Evaluation (with what results?): experiences, perceptions, facilitators, inhibitors.”

### Search strategy

A systematic electronic databases search was conducted during January 2022 for all English articles in six databases, including Pubmed, Web of Science, Cochrane, Embase, CINAHL (EBSCO), and PsycINFO (Ovid). There were no limitations on the year of publication. Searches were based on a combination of free-text keywords and indexed terms (MeSH) related to the terms: aged, elderly, chronic disease, transitional care, patient discharge, patient transfer, continuity of patient care, discharge, discharged home, transition, hospital-to-home, and qualitative research etc. (See Supplementary File 1 for search strategy).

### Inclusion/exclusion criteria

Inclusion criteria: qualitative studies or mixed studies for which qualitative data could be extracted were included, including but not limited to phenomenological, root theory, and ethnographic studies; focus on multiple chronic conditions rather than one specific condition, as studies focusing on a single condition are too specific and not necessarily generalizable to older adults with multiple chronic conditions; all studies included older adults who were at least 60 years of age, as this is the World Health Organization definition of older adults; published in English.

Exclusion criteria: focus on the overall hospital/discharge experience rather than the transition experience from hospital to home; transition to a nursing home or specialized palliative care facility or rehabilitation center; conference proceedings or abstracts, review articles, editorials, clinical case reports, or review articles; articles collected using qualitative methods but analyzed using quantitative analysis; non-English language literature.

**Figure 1:**
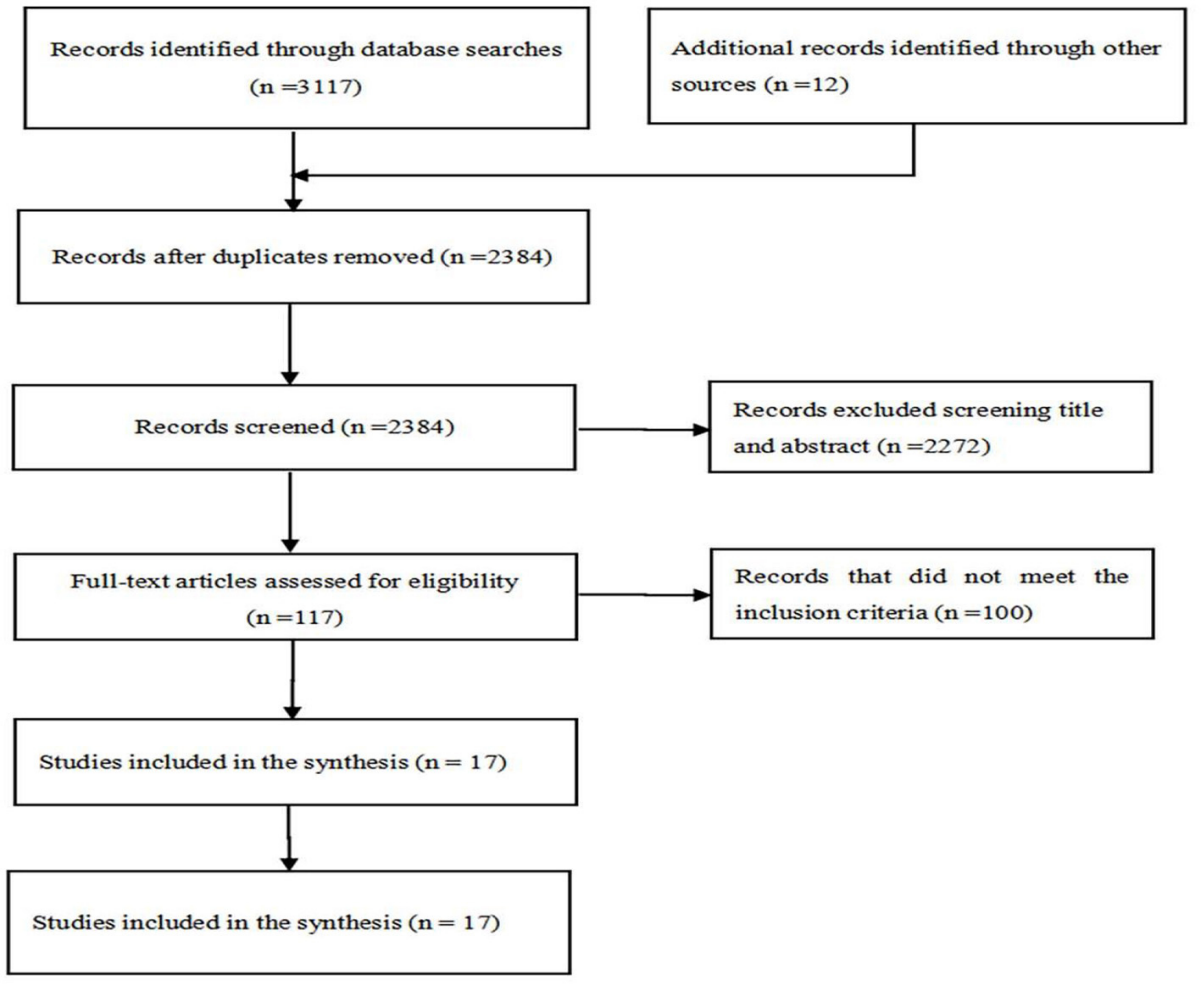
PRISMA flow diagram.

An initial search yielded a total of 3,129 articles. Two researchers independently screened and extracted literature by inclusion and exclusion criteria. The remaining 2,384 articles after removing duplicate literature. In addition, 117 papers remained after reading the titles and abstracts and 100 papers were deleted after reading the full text, for a total of 17 included studies. Seventeen papers were not deleted after quality assessment. The PRISMA flow chart was used in this process (Figure 1).

### Quality appraisal

The Qualitative studies were appraised using The Critical Appraisal Skills Programme (2013) qualitative checklist comprising 10 items relating to rigor, credibility and relevance of qualitative studies (35). All items were scored as “yes” or “no” or “can't tell.” Studies were scored as “high” or “medium” or “low” quality. Quality evaluations were performed by two independent authors, and when disagreements were encountered, a third author moderated. The final evaluation results are shown in Table 1.

**Table 1:** Evaluation of methodological quality.

**Included studies**	**Questions**										**Quality appraisal indicator**
	①	②	③	④	⑤	⑥	⑦	⑧	⑨	⑩	
Dolu et al. (24)	Y	Y	Y	Y	Y	Y	Y	Y	Y	Y	H
Dolu et al. (25)	Y	Y	Y	Y	Y	?	Y	Y	Y	Y	M
Neiterman et al. (38)	Y	Y	Y	Y	Y	?	Y	Y	Y	Y	M
Bull (41)	Y	Y	Y	Y	Y	?	?	Y	Y	Y	M
Bull and Jervis (42)	Y	Y	Y	Y	Y	?	Y	Y	Y	Y	M
Backman et al. (43)	Y	Y	Y	Y	Y	?	Y	Y	Y	Y	M
Allen et al. (4)	Y	Y	Y	Y	Y	?	Y	Y	Y	Y	M
La Manna et al. (39)	Y	Y	Y	Y	Y	Y	Y	Y	Y	Y	H
Graham et al. (49)	Y	Y	Y	Y	Y	?	?	Y	Y	Y	M
Backman and Cho-Young (46)	Y	Y	Y	Y	Y	?	Y	Y	Y	Y	M
Davis et al. (50)	Y	Y	Y	Y	Y	?	Y	Y	Y	Y	M
Hvalvik and Reierson (48)	Y	Y	Y	Y	Y	?	Y	Y	Y	Y	M
McKeown (45)	Y	Y	Y	Y	Y	?	Y	Y	Y	Y	M
Plank et al. (47)	Y	Y	Y	Y	Y	?	Y	Y	Y	Y	M
Baxter et al. (44)	Y	Y	Y	Y	Y	?	Y	Y	Y	Y	M
Foust et al. (26)	Y	Y	Y	Y	Y	?	Y	Y	Y	Y	M
Nikbakht-Nasrabadi et al. (40)	Y	Y	Y	Y	Y	?	Y	Y	Y	Y	M

Y, Yes; N, No; ?, Can't tell. Quality appraisal indicator—“H” = High, “M” = Medium, “L” = Low. Adapted from Critical Appraisal Skills Programme (2013). ①: Was there a clear statement of the aims of the research? ②: Is a qualitative methodology appropriate? ③: Was the research design appropriate to address the aims of the research? ④: Was the recruitment strategy appropriate to the aims of the research? ⑤: Was the data collected in a way that addressed the research issue? ⑥: Has the relationship between researcher and participants been adequately considered? ⑦: Have ethical issues been taken into consideration? ⑧: Was the data analysis sufficiently rigorous? ⑨: Is there a clear statement of findings? ⑩: How valuable is the research?.

### Data extraction and synthesis

The study data extracted were: author, publication year, country, design, setting and sampling, method(s) of data collection, data analysis strategy and aims. Study findings were extracted from the findings/results section of each paper. Thematic synthesis was conducted using a three-stage process (36). Stage 1: the coding of the selected studies text line-by-line. Stage 2: a review of the coding with grouping to generate descriptive themes and subthemes. We drew upon Meleis's Transition theory (37) for data a narrative synthesis, specifically, the domain “transition conditions” to classify potential facilitators and inhibitors as personal, community or societal. Stage 3: the generation of abstract themes or analytic themes.

## Results

A total of 17 studies were included, with studies from a total of nine countries: the United States (*n* = 7), Turkey (*n* = 2), Canada (*n* = 2), Australia (*n* = 1), Tran (*n* = 1), Norway (*n* = 1), Italy (*n* = 1), the United Kingdom (*n* = 1), and Ireland (*n* = 1). Six of the studies had theoretical or conceptual frameworks involving active recovery models, social-ecological perspectives, transition theory, social-ecological models of health behavior, positive deviance frameworks, and constructivism. The sample of participants varied across studies. Eleven of the 17 studies included older adults, 12 studies investigated the perspectives of their caregivers, and 3 studies explored the perspectives of health care providers. Each study was systematically assessed for study purpose, study methodology, sample size, study setting, and country in which the study occurred (Table 2). The methodological quality of the included studies was variable, with 2 being rated as high and 15 as moderate. None of the studies were excluded due to study quality issues.

**Table 2:** Summary of included studies.

**Author**	**Population and setting**	**Sample**	**Design**	**Aim (s)**
Dolu et al. (24) 2021 Turkey	Healthcare providers. Both public and private community healthcare sectors in an urban area of Turkey.	*N* = 13 (5 general physicians and 8 nurses) The average age of healthcare staff was 41.6 years.	Qualitative study, purposive sampling, In-depth semi structured interviews, thematic analysis	To explore the perspectives of healthcare providers, including nurses and physicians, regarding transitional care from hospital to home in an urban area of Turkey
Dolu et al. (25) 2021 Turkey	Older patients and family caregivers. Patients' home.	*N* = 25 (14 older patients and 11 family caregivers) The mean age of the older adults was 79 years.	Qualitative exploratory descriptive study, purposive sampling, in-depth semi-structured interviews, thematic analysis, proactive rehabilitation model	To explore the perspectives of patients aged 65 years and over and their family caregivers transitioning from hospital to home in an urban area of Turkey.
Neiterman et al. (38) 2015 Canada	Older patients and family caregivers. Patients' home.	*N* = 36 (17 older patients and 19 family caregivers) The mean age of the older adults was 79 years.	Qualitative study, purposive sampling, semi-structured interviews, thematic analysis	To examine how the care transition was experienced, organized, and coordinated by patients and their informal caregivers at home.
Bull (41) 1992 America	Older adults and family members. Patients' homes.	*N* = 55 (17 older patients and 19 family caregivers) The mean age of the older adults was 67 years.	Qualitative study, purposive sampling, semi-structured interview, contant comparative method	To describes the period of transition from hospital to home based on the perspectives of the older adults and family members who experience it
Bull and Jervis (42) 1997 America	Older women and caregiving daughter. Participants' homes and private offices.	33 mother-daughter pairs at 2 weeks post-discharge (response rate 94%) and 32 pairs at 2 months post-discharge. The average age of the mothers was 73.9 years.	Qualitative study, grounded theory study, purposive sampling, semi-structured interviews, content analysis and constant comparison	To learn how older women and their caregiving daughters managed care following the mother's hospitalization because of a chronic illness.
Backman et al. (43) 2018 Canada	Patients with multiple chronic conditions and family members. Patients' home.	*N* = 9(4 older adults alone, 3 family members alone, and 2 older adult/family member together) Mean age of 77.6 years.	Descriptive qualitative study, participatory visual narrative methods, convenience sampling, thematic analysis, socio-ecological perspective	To engage older adults with multiple chronic conditions and their family members in the detailed exploration of their experiences during transitions across health care settings and identify potential areas for future interventions.
Allen et al. (4) 2018 Australian	Patients and caregivers. Patients' home.	*N* = 26 (19 Patients and 7 carers) Participants were aged on average 78.9 years.	Descriptive qualitative study, purposive sampling, semi-structured interviews, thematic analysis, constructivism	How do older people and their carers/families as care recipient service users, experience discharge and transitional care across the trajectories of acute, subacute and community care?
La Manna et al. (39) 2018 America	Older Patients. Patients' home.	*N* = 96 (Multimorbidity was prevalent among study participants) Mean age of 75.15 years.	Quantitative-qualitative, mixed-method design, inductive content analysis techniques, transition theory	To examine self-described hospital-to-home transition challenges encountered by older adults with a diagnosis of diabetes within the first 30 days following discharge
Graham et al. (49) 2009 America	Older person and cares. NR	Twenty focus groups (*n* = 159 caregivers included family members, friends, and paid informal caregivers) and focus group (*n* = 5, The five seniors selected were age 60 or older).	Qualitative study, purposive sampling, qualitative thematic analysis, social ecological model of health behavior	To assess the needs of patients and caregivers during the transition from hospital to home.
Backman and Cho-Young (46) 2019 America	Patients and informal caregivers. NR	*N* = 8 (Patients and informal caregivers). Mean age of 72.29 years.	Qualitative descriptive study semi-structured telephone interviews, convenience sampling, thematic analysis	To describe patients and informal caregivers' perspectives on how to improve and monitor care during transitions from hospital to home
Davis et al. (50) 2012 America	Health care professionals, clinicians, care teams, and administrators from the inpatient general medicine services at one urban, academic hospital; Two outpatient primary care clinics.	13 focus groups and two in-depth interviews with 75 health care professionals and administrators. The average age of healthcare staff was 42 years.	Cross-sectional qualitative study, purposive sampling, thematic analysis	To understand care transitions from the perspective of diverse healthcare professionals, and identify recommendations for process improvement.
Hvalvik and Reierson (48) 2015 Norway	The next of kin to an older patient (age 67 and older):such as spouse, sons, or daughters. NR	*N* = 11 (one son, two spouses, and eight daughters)	Phenomenological hermeneutic design, individual, narrative interviews, purposive sampling	To describe and illuminate the meaning of the next of kin's lived experiences during the transition of an older person with continuing care needs from hospital to home.
McKeown (45) 2007 Ireland	Older people (in their homes two weeks following discharge from acute hospital). Patients' home.	*N* = 11 (6 males and 5 females) The age of elderly entry ranged from 71–92 years old.	Qualitative study, phenomenological approach, semi-structured interview, purposive sampling	To explore the experiences of older people on discharge from hospital following assessment by the public health nurse
Plank et al. (47) 2012 Italy	Informal caregivers. Rehabilitation unit.	*N* = 18 (8 participating in individual interviews and 10 participating in post discharge focus groups, three caregivers attended both the interview and the focus group)	Qualitative phenome nological approach, in-depth interview, focus group, purposive sampling	To explore and understand the experience of new informal caregivers in Italy in the time of transition from hospital to home, focusing on their thoughts and reflections.
Baxter et al. (44) 2020 UK	Multidisciplinary staff. Six high-performing general practices and hospital specialtie.	*N* = 157(including doctors, nurses, healthcare assistants, allied health professionals, discharge coordinators, district nurses, community matrons, specialist nurses and receptionists/administrators).	Qualitative study, focus groups, interviews and brief observations, in semi-structured focus groups or interviews and meetings, opportunity and maximum variation purposive sampling, pen portrait approach, positive deviance framework	To explore how high-performing general practice and hospital teams successfully deliver safe care to older adults during transitions from hospital to home
Foust et al. (26) 2011 America	Older adults, carers, health providers. Patients' home and hospital	*N* = 90 (40 patients, 35 informal caregivers, and 15 clinicians). The mean age of patients was 64.8 years.	Descriptive qualitative, semi-structured Interview, content analysis, purposive sample	To describe the hospital-to-home transition from the three perspectives of home health patients, their informal caregivers, and home health care clinicians
Nikbakht-Nasrabadi et al. (40) 2021 Iran	Family caregivers. Either in their home or other else	*N* = 15 (family caregivers of patients with multiple chronic conditions).	Descriptive exploratory qualitative study, in-depth semi-structured, face-to-face interviews, purposive sample, content analysis	To explore the experiences of family caregivers of transitional care in diabetes with concurrent chronic conditions

In this review, Personal and community focused facilitators and inhibitors were identified that mapped to there themes: (1) Resilience in older adults; (2) Interpersonal connections and relationships; (3) Uninterrupted transfer of care supply chain (Table 3). These facilitators and inhibitors existed at the health care provider level, at the patient and family level, and at the health system level.

**Table 3:** Transition facilitators and inhibitors.

**Theme**	**Transition conditions PC = Personal conditions, CC = Community conditions, SC = Societal conditions**	**References**
Resilience in older adults;	**Facilitators**	
	• Coping strategies (PC): such as - Acting on past experience - Creating a schedule, adapting the home environment - Integrating effective information - Learning self-care skills	(4, 25, 41–43)
	• Personal Traits (PC):positive, cooperative	(4, 43, 44)
	**Inhibitors**	
	• Negative perception transition (PC)	(25, 39, 41, 43, 46)
	• Knowledge gap in managing symptoms (PC)	(39, 46)
Interpersonal connections and relationships		
Patient-caregiver connections and relationships	**Facilitators**	
	• A positive, caring relationship (CC)	(4, 43, 46)
	• Family caregivers actively empower, advocate, and motivate patients (CC): - Practical support e.g., life care, professional care - Emotional support e.g., courage, willingness to care	(4, 25, 41–43, 45, 47, 48)
	• Cultural concept of filial piety (CC)	(49)
	**Inhibitors**	
	• Residence status: especially widowed elderly living alone (PC)	(38, 45)
	• Physical and mental symptoms and financial burden of caregivers (PC)	(39, 40)
Patient/caregiver-healthcare provider connections and relationships	**Facilitators**	
	• Healthcare providers making efforts to understand patients (CC)	(44)
	• Caring from a healthcare providers (CC)	(4)
	• Patients and families participate in care decisions together (CC)	(4, 43)
	• Setting Navigator/Transitional Care Coordinator (CC)	(38, 46)
	**Inhibitors**	
	• Indifferent tone and attitude of health care providers (PC)	(40)
	• Use of terminology in conversation (PC)	(25, 26)
	• Ignore identity presentation and interaction (CC)	(4)
	• Organizational factors: such as specific time and workload constraints (CC)	(25, 43)
	• E-health literacy (CC)	(50)
Connections an relationships between health care providers	**Facilitators**	
	• Regular meetings (CC)	(44, 50)
	• Mutual trust between healthcare providers to support or quickly respond to requests (CC)	(44)
	• Communication media e.g., letters, electronic medical records or digital calls supported by technology (CC)	(46)
	**Inhibitors**	
	• Employee rotation and reorganization (CC)	(44, 50)
	• Hospitals and community organizations are independent (CC)	(44, 50)
	• Communication media e.g., letters, electronic medical records or digital calls supported by technology (CC)	(50)
Uninterrupted transfer of care supply chain	**Facilitators**	
	• Care coordination practices (CC), for example - Discharge coordinator: transition nurse - Multidisciplinary team to reach consensus on care transition delivery through meetings. - Patient and family participation in decision making, monitoring or supplementing the care transition process. - well-developed electronic systems and written information from a holistic perspective	(25, 38, 43, 44, 46, 47, 49)
	**Inhibitors**	
	• Lack of standardized processes and fragmented communication (CC)	(43, 46, 50)
	• Different positioning of healthcare providers (CC)	(44, 50)
	• Gaps in discharge planning (CC): handover of discharge t information	(24, 26, 46, 47, 50)
	• Approaches to care (CC), such as - Care provider-centered care - Organization of care (e.g., organizational responsibilities are not clearly defined)	(4, 38, 40, 43, 50)
	• Human resource limitations (CC), for example - Inadequate staffing - Insufficient staff knowledge and skill level - Family caregiver avoidance and misconceptions about caregiving responsibilities	(24, 38, 45, 47, 49)
	• Patients' residential distance (CC),	(45, 49)
	• Adherence to healthcare authority (PC)	(25)

### Resilience in older adults

Resilience in older adults refers to the ability of the elderly to accept the transition from hospital to home and to make sense of the benefits and losses associated with the transition. We identified several facilitators and barriers that were compatible with Meleis's personal and community transition conditions.

For older adults, the perceived benefits of going home include access to comfort, freedom (e.g., following one's own opinions in daily life without the constraints and supervision of organizations), safety (avoiding hospital-acquired infectious risks), and a sense of personal control (4, 25, 38). Care transitions can be a difficult and unsafe time for most participants (25, 38–40). Resilience as a force against adversity involves positive thinking, lifestyle changes and engagement in self-management to readjust within an unstructured family environment, become independent and re-establish new routines (4, 25, 41–43). Adapting to a new environment at home and achieving a safe transition seem to benefit from personal traits such as motivation, initiative, and cooperation (4, 43, 44). One contributing factor was the personal belief that older adults want to be independent, which motivated them to find a personal strategy and solution to the dilemma of care transition and dependency.

During the care transition, older adults had difficulties with self-management in daily living, professional care and follow-up care, such as dressing, eating, bathing and transportation, medications, wounds, follow-up visits (4, 24, 38, 39, 41, 42, 45). Potential facilitators of transition were reinvention strategies used by older adults to meet independence and personal care needs, such as self-motivation, acting on previous experiences, developing schedules, engaging in activities of daily living and co-learning, proactively seeking help from others, integrating valid information, and mastering self-care skills (e.g., complex medication management) (4, 25, 41–43).

Inhibiting factors were negative perceived transitions as discontinuity and powerlessness, loss (25, 39, 41, 43, 46), which were related to the patient's health status, roles and relationships, daily life and hobbies, and gaps in self-care knowledge. Fear of leaving the hospital exacerbates disease progression in older adults due to unresolved physical symptoms (25). Patients with delayed self-recovery feel depressed and frustrated (39). Older adults presented a strong care dependency during transition. Participants indicated that maintaining daily life and managing complex health issues after returning home was a challenge (4, 24, 38, 39, 41, 42, 45) and that daily management and hobbies required forced changes (38, 41, 45). Gaps in knowledge when dealing with physical changes and adverse reactions can leave participants will be in a difficult situation (38, 39, 46).

### Interpersonal connections and relationships

Interpersonal relationships and connections among older adults concentrated among family caregivers (including spouses, children, neighbors, friends, etc.), and health care providers. We identified several facilitators and barriers that were compatible with Meleis's personal and community transition conditions.

Patient-caregiver connections and relationships: a positive and strong relationship with caregivers was an important factor for a smooth transition for older patients. Caregivers actively empowered, advocated, and motivated patients in care transitions (4, 41–43, 45, 47, 48), including closely monitoring older adults' health status; facilitating communication between older adults and health care providers; helping older adults improve their self-management; and focusing on the care delivery process to ensure a smooth transition. In addition, caregivers provided emotional support, such as hope, courage, and willingness to enter the caregiver role, so that patients felt dependent and confident as well as unabandoned (4, 43, 47). In some cultural settings, the value of filial piety became a double-edged sword and caregivers and patients had to live together. Cultural values and practices that do not allow elderly people to be sent to nursing homes constitute, at another level, a barrier to the use of formal care (49).

Caregivers experienced physical and emotional and financial stress when confronted with the complexity and number of chronic illnesses suffered by family members, combined with their own physical condition and work and patient cooperation, which may result in burnout and avoidance (39, 40). In addition, An important inhibiting factor was the patient's residential status (38, 45). Older adults who were widowed, especially those who live alone, were vulnerable to the loss of basic social relationships and connections, which not only exacerbated depression and isolation (45); they also tended to miss out on the symptom monitoring and care support provided by their families and put themselves at a disadvantage (38).

Interpersonal relationships and connections between older patients and families and healthcare providers was identified as potential facilitators and inhibitors of health transitions (4, 25, 38, 43, 44, 48). The nature of the relationship between older adults and health care providers was diverse and was described as collaborative and supportive (43), caring (4), trusting rapport (44), and skeptical (47, 48). Promoting meaningful patient/caregiver-provider relationships was a way for healthcare providers proactively spend more time with patients or families in an effort to understand the health status and care needs (44); to involve patients and families in healthcare decisions (4); to conduct regular follow-up visits (46); and, especially in cross-border situations, to establish intermediaries to navigate relationships, organize contacts to ensure patients know when problems arise whom to contact (38, 46).

Inhibiting factors were included: indifferent tone and attitude of medical provision (40); use of medical jargon in conversations; giving verbal information that may be forgotten and written information that is not readable (25, 26); neglected identity presentations and interactions (4); poor previous care and medical records (47); and organizational factors such as specific working hours and workload limitations (25, 43). A potential inhibiting factor lay in electronic communication devices (50). Older adults with limited e-health literacy had difficulty staying in communication with health professionals in digitally supported conversation and appointment systems and felt separated and alienated from their interpersonal relationships.

Establishing or maintaining valuable relationships among health care providers appeared to be important in facilitating the transition of older adults (44, 45). One barrier factor was the instability of relationships between healthcare providers, which was a matter of systems and organizational processes, such as regular staff rotation and reorganization, which meant that old familiar partnerships were broken up and had to be regrouped; the independence of hospitals and community health organizations from each other lacking feedback on patient transition status, channels for sharing and opportunities for cross-border learning, which reduced interpersonal interactions and contacts among healthcare providers and thus may not allow for timely tracking of patient transition status (44, 50). Communication media such as letters, electronic medical records, or digital calls supported by technology were considered potential facilitators or inhibitors that helped facilitate valuable contact between healthcare providers regarding patient follow-up care services (46) or relyed solely on electronic cases to convey information or discharge processes, neglecting interpersonal communication (50). Trust was a cornerstone of many relationships among healthcare teams and an important facilitator of maintaining connections. Mutual support or rapid response to requests among healthcare members across settings helps strengthen relationships and connections (44). Regular meetings brought members of teams together, and such multidisciplinary meetings provided opportunities for socialization and interaction, not only to maintain contact and relationships, but also to support patients through verbal communication (44, 50).

### Uninterrupted transfer of care supply chain

The theme of uninterrupted transfer of care supply chain centered on care coordination, approaches to care, and workforce factors. Potential facilitators and inhibitors corresponded with Meleis's personal and community transition conditions.

Care coordination was a facilitator of hospital-home care delivery for patients (25, 38, 43, 44, 46, 47, 49). An important manifestation of care coordination was the discharge coordinator, who was the central point of contact between the hospital and the community agency or family physician or general practitioner, especially the transition nurse, who was familiar with the way the health system works and provided a liaison for care coordination across hospitals/communities (25, 38, 43, 44, 46, 49). Whereas, communication was considered an important means of care coordination, multidisciplinary teams share information seamlessly between different team members and/or specialists through formal or informal meetings, raised relevant issues, and set transition goals and priorities to reach consensus on care delivery to avoid inconsistency and loss of trust (47). Other facilitators included involving patients and families in discharge planning decisions and discussions, the ability of patients and families to add and share more nuanced information as they move across health care settings to help care providers better understand the situation and make decisions (25, 46), or monitoring the entire care delivery process so that information was not misplaced (46, 48). Potential facilitators included well-developed electronic systems and written information with a holistic perspective so that staff in care transition delivery act on established plans and relevant symptom management knowledge and information (24).

Factors impeding care coordination included: lack of standardized processes and fragmented communication (43, 46, 50) differences in the positioning of hospital and community care providers' roles and thus priorities and goals, and possible conflicting values and understandings that impede care coordination (44, 50); other inhibiting factors included: gaps in discharge planning, where patients and caregivers were often passive and hastily accepted discharge plans (26); arbitrary and incomplete handover of discharge information, e.g., care provides giving verbal information that may be forgotten, and primary care providers or family physicians do not receive or delay receiving or receive incorrect, unclear discharge information or letters (24, 26, 46, 47, 50).

Approaches to care had the potential to inhibit transition. This included: approaches to care that promoted resident care dependence rather than greater patient self-management (4); care provider-centered care that did not take into account patient preferences and uniqueness (40, 43); and care organization (e.g., organizational responsibilities were not clearly defined (50), multiple care providers emerged in a confusing manner after discharge, and patients did not know who to contact when problems arose (38).

A potential inhibiting factor was human resources. With inadequate staffing, patients felt abandoned and helpless by the organization as they were unable to receive accurate care and assistance from medical staff at the right time or when they needed it most (38, 45). The limited level of knowledge and skills in the hospital-to-home delivery process, e.g., home health care providers were powerless in the face of new medical equipment due to gaps in continuing education (24). And family caregivers' ambiguous answers to questions about symptoms, questioning their ability to provide home care, and thus avoiding responsibility for home care or the misperception that it should be borne entirely by the health care provider, were often associated with limited discharge planning (24, 47).

In addition, other inhibiting factors including the distance patients live (45, 49) and adherence to medical authority (25) also hindered care delivery. If older adults were afraid to question care delivery methods and questions or live far from hospitals, communities, or in rural areas where there was a lack of primary care organizations, the lack of continuous supply of care services may affect patient transition.

## Discussions

The ideal outcome for older adults transitioning from hospital to home is a healthy transition in which the physical, psychological, and emotional needs of older adults with follow-up care services are met and independence is gradually achieved. Our systematic review identified factors that may facilitate or inhibit healthy transitions from hospital to home for older adults, with implications for the development of transitional care services. Facilitators and barriers were mapped to three themes with an individual and community focus: Resilience in older adults; Interpersonal connections and relationships; Uninterrupted transfer of care supply chain. These themes resonate with the broader international literature (30, 51–54) on the losses and gains of care transitions (51, 52), elements that reduce readmission rates for older patients with chronic conditions (30, 53), and competencies needed for transitional care providers (54), such as: education and promotion of self-management, maintenance of relationships and promotion of coordination, communication and health care provider training.

For the topic of resilience in older adults, person-centered transition facilitators included older adults' values, positive personal attributes, and personal coping strategies in the face of self-management barriers. Chronic illness is a problem-based endeavor in which participants, with limited medical resources, often act on previous experiences, struggle to seek help, and employ self-management strategies to deal with health issues. Older adults want to be as independent as possible and stay at home for as long as possible. Personal strategies driven by beliefs to cope with self-management barriers emerge to make a smooth transition possible. Previous research had also found that older adults were struggling to find ways to master new situations that were useful and not burdensome for others (55). However, in the actual transition, there is a gap between the self-management performed by older adults and the self-management imagined in the discharge instructions (56). The lack of knowledge and self-management skills led to uncertainty and anxiety in some participants. In addition, unresolved somatic symptoms, delayed recovery from self-care, difficulties in daily management and processing of information, and inadequate social support also had an impact on patients' psychological mood. These physically, psychologically, and socially diverse factors contribute to the uncertainty and powerlessness of patients' transitions. This is consistent with studies by Joo et al. (27) and Hardy et al. (57). MacLeod et al. (58) reported that resilience interventions currently designed for older adults are often not available *per se*, but seem to show life for resilience building by improving adaptive coping, changing complex emotional responses (e.g., anxiety, depression), and social support. Patients with chronic illness often need knowledge in order to objectively interpret the illness and cope effectively. In this context, patient education before discharge and intensive self-management training after discharge seem to facilitate patients' adaptation to the transition (30, 59, 60). Furthermore, in the theme of uninterrupted care supply transfer chain we mentioned the impediment of caregiver (family caregivers and medical caregivers) incompetence to care transition. Therefore, in addition to patients, formal and informal caregivers need to develop competencies to help patients with self-management education and training.

The two mindsets of older chronically ill patients regarding care transition gains and losses are intertwined and constantly dynamic. Participants can be seen to have gained some important developments in their care transitions, such as: regaining independence, sense of personal control, etc. However, unsurprisingly, some participants appeared to experience fewer gains, and their journeys contained more pronounced losses than others, reporting strong feelings of anxiety, depression, and becoming barriers to transition. Notably, although psychological problems such as anxiety and depression are common in patients after discharge, screening is not routinely performed at discharge. Common outcome indicators for most transitional care interventions remain objective indicators such as readmission rates and mortality (61), with limited attention to patient-related outcomes, such as complex emotional problems. A prospective study reported (62) that hospital-home transition, as a period when older adults are at high risk for depressive symptoms, screening for identification of depressive symptoms and assessment of coping skills at discharge may be a potentially preventive intervention. Esche et al. (52) showed that resilience, as a factor that must be considered when transitioning older adults from hospital home, requires early identification by nurses and help those patients with low resilience to succeed in the home environment. Thus, these facilitators and barriers have the potential to inform the development of resilience-specific interventions to promote the psychological, social, and physical wellbeing of older patients with chronic conditions during transitions of care. Furthermore, intervention development has to consider the potential contribution of conceptual models such as Meleis' transition theory, the active recovery model, and the social ecology of health promotion.

Connections and relationships between the elderly and their caregivers, healthcare providers, and healthcare workers play an important role in facilitating and inhibiting patient transition and engagement in care. Our study suggests that patient and family caregiver partnerships that fill supply gaps, navigate the health care system, and advocate on behalf of patients can improve the health wellbeing of older adults. Findings also highlight that contextual factors (e.g., cultural attributes, roles and responsibilities of family members, residential status, etc.) must be considered to support these important relationships. There is a link between interpersonal continuity and patient satisfaction with health care (63). The medical staff can act as a barrier or facilitator, depending on his communication skills and ability to involve patients in decision making. Our study showed that a cordial, trusting relationship with the provider was an important factor in facilitating transition and supporting patient involvement, which is consistent with a study by Stolee et al. (64), which reported similar characteristics of a beneficial patient-provider relationship, including trust and respect, and guided patient and family involvement in decision making related to treatment during the transition. Moreover, a study by Mitchell et al. found that cold, brief discourse was detrimental to the establishment and maintenance of the relationship. This is consistent with our study's findings. To facilitate communication and connection and improve relationships with healthcare professionals, information communicated verbally and in writing must be shared in language that is jargon-free and easily understood by service users and caregivers. Health care workers need biopsychosocial training including communication skills, the need to consider the risk of giving inadequate or marginalized elderly wellness, and the need to assess the ability and understanding of older adults to understand information to ensure that both parties are working at a uniform level and to correct inequalities in power between service users and providers, which is particularly important in the new person-centered care model promulgated by WHO and other health agencies (65, 66).

Our study also highlights the importance of maintaining relationships and connections among HCPs. Previous studies have shown that healthcare providers establish cross-border relationships that can overcome discontinuous letters and uncertainty at transition (67). Healthcare providers have also indicated that regular meeting interactions (formal or informal) provide opportunities and channels for knowledge sharing, information feedback, and interpersonal communication that not only help healthcare providers gain knowledge of each other's work environment and develop interpersonal relationships, but also build trust and mutual understanding of each other's roles of responsibility, thereby maintaining organizational stability and continuity of patient care treatment during hospital-to-home transitions and consistency of patient care during the hospital-to-home transition (68, 69). Participants perceived that healthcare professionals build trusting relationships that allow for rapid response to each other during care transitions. A previous study by Bryn et al. (70) demonstrated little effective tools to support trust among HCPs when collaborating across borders when in different locations and lacking shared electronic health records, and at the broader health care system level. Baxter et al. (44) suggested that supporting staff to build and maintain relationships should be prioritized from an organizational systems perspective, such as minimizing staff rotation or having the same values and beliefs among staff.

Transitions are uncertain and complex (37), and the transition of older adults from hospital to home is not a linear process, but one of cross-cultural challenges for two organizations with different values and priorities. Some participants indicated that the different positioning and nature of services between hospitals and community agencies influence Healthcare workers' perceptions, expectations, and priorities for care transitions (25, 44, 50). Healthcare professionals need to be familiar with each other's roles and functions and clearly position themselves to minimize professional, cultural, and organizational differences (71). In fact, care plans and instructions between providers still conflict with each other. Previously Naylor et al.'s (11) transitional care model utilized transitional care coaches to facilitate care coordination through the advanced practice nurse and Coleman et al.'s (60) transitional care intervention model. Participants (25, 38, 43, 44, 46, 49) also reported that roles in the nursing process can facilitate care transitions and ensure an uninterrupted supply chain of care delivery. The importance of nurses as core healthcare practitioners and facilitators of transitional care continues to emerge. This is due to the professional status of nurses and their close interaction with patients, their familiarity with the way health systems work, and their leadership in collaboration. In the past few years, several Meta-analyses have shown that nurse-led transitional care, which respects patients' independence and decision-making power in care, is effective in increasing satisfaction, improving health status, reducing readmission rates, and is cost-effective and economically efficient (72, 73). Despite these developments, the inclusion of 17 studies in our review, only 3 of which investigated the views of medical caregivers, and no qualitative studies of transition nurses have been included, is a weakness of the existing literature. This supports our call for all stakeholders to be involved. Future research could therefore explore how nurses face barriers and experiences in collaborating across organizations.

### Limitation

A systematic review of multiple perspectives of older patients, caregivers, and health professionals using Meleis' transition theory constructed our conceptualization of situational transitions and helped us classify facilitative and barrier factors with the help of the transition condition domain. However, we only included studies published in English in our review, so we may have missed relevant studies from non-English speaking countries, a potential language bias; there was a little theoretical framework in the studies and the timeline of the transition process varied, with some studies collected from the day patients were discharged and some 1 month or more after, and the heterogeneity of patient experiences and feelings limits the generalizability of the study to some extent. Finally, as this was a secondary analysis, we were unable to confirm our themes and consequences with the study participants.

## Implications for practice

Identify individual and community-focused facilitators and inhibitors to inform the development of interventions to promote healthy transitions in older adults.

- Assess the psychological status of older adults at discharge in addition to the physical status of the patient.
- Healthcare staff need to improve capacity in addition to enhancing patient and family education.
- Build trusting relationships between health care providers, professionals, and older adults and their caregivers.
- Further explore the perceptions of health care workers, especially transition nurses, regarding transitions of care in order to improve transition care at the organizational system level.

## Conclusions

This study identified potential transition facilitators and inhibitors for incoming older adults transitioning from hospital to home, and these findings may inform the development of interventions to target key areas of resilience in adapting to a new home environment, and human relations and connections for building partnerships, as well as an uninterrupted supply chain of care transfer at hospital-home delivery.

## Data availability statement

The original contributions presented in the study are included in the article/Supplementary material, further inquiries can be directed to the corresponding author.

## Author contributions

MS and JW were responsible for the conception, design of the review, literature search and screening, analysis and interpretation of data, and drafted the manuscript. LL contributed to the data acquisition and interpretation and revised the paper. MZ, TX, and YQ contributed to data analysis and interpretation. RD contributed to the design of the review and revised of the manuscript. All authors approved the final version of the manuscript for submission and the type of the magazine.
