# Supplementary material for: Facilitators and inhibitors in hospital-to-home transitional care for elderly patients with chronic diseases: A meta-synthesis of qualitative studies
Source: Front Public Health. 2023 Feb 13;11:1047723. doi: 10.3389/fpubh.2023.1047723 (PMC9969141; doi:10.3389/fpubh.2023.1047723)
Supplement: Supplementary file 1 [file Table_1.DOCX]

**Take Embase for example**

#1 'aged'/exp or aged:ab,ti OR elder*:ab,ti OR geriatric*:ab,ti OR senior*:ab,ti OR old*:ab,ti OR 'older adults':ab,ti OR 'older people':ab,ti OR 'older patient':ab,ti OR 'old age':ab,ti OR 'old person':ab,ti OR 'aging adult*':ab,ti OR 'ageing adult*':ab,ti

#2 'chronic disease'/exp or 'chronic disease*':ab,ti OR 'non infectious disease*':ab,ti OR 'non communicable disease*':ab,ti OR 'chronic non-infectious disease*':ab,ti OR 'non-communicable disease*':ab,ti

#3 'transitional care'/exp OR 'patient care'/exp OR 'hospital discharge'/exp OR 'patient transport'/exp OR 'transitional care':ab,ti OR 'transition care':ab,ti OR 'care transition':ab,ti OR 'patient transfer':ab,ti OR 'patient discharg*':ab,ti OR 'discharge plan*':ab,ti OR 'continuity of care':ab,ti OR 'continuous care':ab,ti OR 'continuum of care':ab,ti OR 'seamless care':ab,ti OR diacharg*:ab,ti OR transition*:ab,ti OR transfer:ab,ti OR continu*:ab,ti

#4 'independent living'/exp OR home*:ab,ti OR communit*:ab,ti OR 'community living':ab,ti OR 'independent living':ab,ti OR 'hospital to home':ab,ti OR 'hospital-to-home':ab,ti OR 'back home':ab,ti OR 'discharged to home':ab,ti OR 'discharged home':ab,ti

#5 'information processing'/exp OR 'qualitative research'/exp OR 'narrative'/exp OR 'interview'/exp OR experience*:ab,ti OR opinion*:ab,ti OR thought*:ab,ti OR attitude*:ab,ti OR subjective:ab,ti OR interview*:ab,ti OR phenomenol*:ab,ti OR feel*:ab,ti OR perspectiv*:ab,ti OR viewpoint:ab,ti OR idea*:ab,ti OR perce*:ab,ti OR understand*:ab,ti

#6 'nursing methodology':ab,ti OR 'case study':ab,ti OR 'constant comparison':ab,ti OR 'content analysis':ab,ti OR 'descriptive study':ab,ti OR 'discourse analysis':ab,ti OR ethnography:ab,ti OR exploratory:ab,ti OR feminist:ab,ti OR 'focus group':ab,ti OR 'grounded theory':ab,ti OR hermeneutic:ab,ti OR interview:ab,ti OR narrative:ab,ti OR naturalistic:ab,ti OR 'participant observation':ab,ti OR phenomenology:ab,ti OR 'qualitative method':ab,ti OR 'qualitative research':ab,ti OR 'qualitative study':ab,ti OR 'thematic analysis':ab,ti

#7 #1and #2 and #3 and #4 and #5 and #6
